# Current Status of Chinese Medical Students’ Professional Identity After COVID-19 and the Factors That Influence It

**DOI:** 10.3389/fpsyg.2022.816767

**Published:** 2022-05-25

**Authors:** Tinghong Lai, Weidong Liang, Maolin Zhong, Pingping Zhu, Bin Li

**Affiliations:** ^1^School of Education, Guangzhou University, Guangzhou, China; ^2^School of Nursing, Gannan Medical University, Ganzhou, China; ^3^First Affiliated Hospital of Gannan Medical University, Ganzhou, China

**Keywords:** medical student, professional identity, COVID-19, status, factor

## Abstract

This study aimed to explore the current situation regarding medical students’ professional identity after COVID-19 in China, as well as the factors that influence it. Questionnaire compiled by Fujian Medical University and self-designed were used, and participators were from one Medical University in Jiangxi Province, a central city of China. Results showed that the professional identity of medical students was upper middle level and the professional attitude was generally positive. There was a significant sex difference in terms of value (*t* = 2.057, *p* < 0.05) which the scores for boys were higher than girls, whereas the scores for girls were higher than boys when it came to aspects such as professional environment (*t* = -3.918, *p* < 0.001) and professional cognition (*t* = -3.855, *p* < 0.001). There was a significant difference in the sense of professional identity between people with and without siblings (*t* = 2.264, *p* < 0.05). The scores of students who participated in prevention and control of the epidemic were significantly higher than those who did not (*t* = 2.267, *p* < 0.01). Professional identity decreased gradually in related to higher grades, but it increased at the graduate stage; the grade [*F _(5,635)_* = 10.302, *p* < 0.001] and majors [*F*_(2,635)_ = 5.718, *p* < 0.01] differences were significant. Factors such as family members’ influence, attitude toward occupation, grades, major, registered residence, and college choosing were the main factors that influenced medical students’ professional identity. Overall, the medical students’ professional identity needs to be further strengthened in the post COVID-19, it should be increased education regarding career development and planning.

## Introduction

### Research Background

According to the statistics of the National Health Commission, there were 4.086 million doctors in China, and the number of doctors per 1,000 people was 2.9 by the end of 2020 (National Health Commossion of the People’s Republic of China, 2020). In 2020, the General Office of the State Council issued the guiding opinions on accelerating the innovative development of medical education, which stated that it was necessary to reform the contents, curriculum, and methods of undergraduate medical education. Its aims included promoting the “excellent doctor education and training plan 2.0,” strengthening the professional quality of medical ethics, and ensuring the integrity of education in scientific research. Other aims were to allow for the ideology and politics of the curriculum to be communicated, to cultivate the spirit of saving lives and healing the wounded, and to treat medical talents benevolently (GBF, 2020). The development of medical students’ talents forms an important part of their career development. Their future careers form part of the goal to realize the Healthy China Strategy.

Professional identity refers to an individual’s positive evaluation of their occupation (Ji et al., 2015). It is a process and a state. It refers to the dynamic and continuous interaction between the individual and the professional environment. As such, there can be differences in people’s professional identity at different times in the process. Professional identity refers to an individual’s overall view of the objectives, social values, and other factors of their occupation; these make up the “basic characteristics” of their occupation (Zhao and Gao, 2020). Medical student’s professional identity encompasses their views on their future career and role as doctors. It is the starting point for a medical students’ personal development and forms a key part of their career development. Given the implementation of new medical reforms, the standardized training system for residents, the deteriorating doctor-patient relationship, the negative role of public opinion, and the dual pressure from the policy system and the social environment, it is worth paying more attention to the professional identity of medical students. A study (Ji et al., 2015) which investigated medical students in four undergraduate medical colleges in Shandong Province found that the overall score of medical student’s professional identity was below average. Under the current system, the medical environment is complex, the public lacks medical knowledge, their expectations are too high, and the trust in doctors is reduced, this can cause disputes between doctors and patients. Public opinion has a subtle impact on both students and interns, which can negatively affect their professional identity. A survey of undergraduate students majoring in clinical medicine at the Medical College of Shantou University (Wu et al., 2017a) found that the professional identity of medical student was at the average level. They also found that there were statistically significant differences between different grades (Wu et al., 2017b). Another survey showed that Chinese medical students generally have a lower sense of professional identity than American students. This is because the improvements in the abilities of Chinese medical students are not reflected in their sense of professional value.

Doctors tend to be physically and mentally exhausted. In general, they are not satisfied with their personal income. They work long hours with a heavy workload, face significant risks and mental pressures, must study and strive to be promoted, have a small basic salary, limited personal safety, and no social status. Moreover, doctors’ working hours and occupational risks increased during COVID-19. COVID-19 has affected the future career choices of more than half (56.8%) of medical students, this impact has been positive for 40.4% students, and negative for 16.2% students, the proportion of junior medical students who have a positive sense of professional identity after COVID-19 is higher than the proportion of senior students in medical college (Li Y. et al., 2020). Otherwise, COVID-19 has a longer incubation period, is more prone to mutation and is more infection, it will cause more serious physical and mental problem. Public health emergency like COVID-19 will influence emotions, young people (aged 15–29 years) are more susceptible to outside influence (Li W. et al., 2020), the working forms and methods of mental health education is weak, which causes the emerging problems of mental health that college students have been confronted (Li, 2020), it is necessary to focus on the change of medical students’ professional emotion.

### Research Purpose

Since the outbreak of the new coronavirus pneumonia (NCP), medical science has been playing a more prominent role in human health care and social development. Improving the quality of medical education and creating high-quality medical talents are important goals for the current era. This raises the following questions: Has the status of medical students’ professional identity changed since the outbreak of COVID-19? What are the influencing factors? How can medical students improve their professional identity? It is benefit to reform departmental teaching and promote the development of medical education to explore those question, it will provide a reference point for the development of medical education.

## Method and Results

### Objects and Method

#### Objects

**FIGURE 1:**
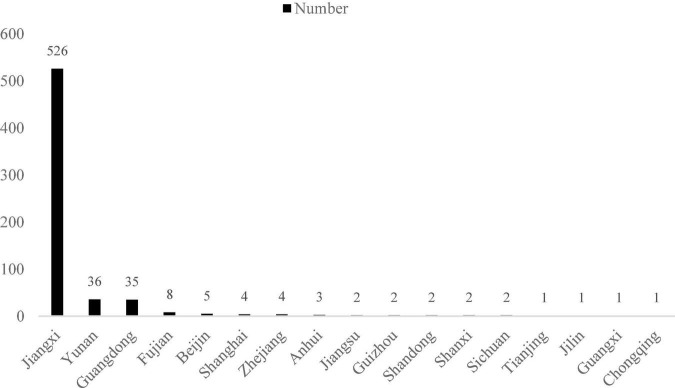
Geographical distribution of medical student in the research.

635 valid questionnaires were distributed in one medical college in Jiangxi Province via the Internet, including 439 (69%) from native and 196 (31%) from the other province (see Figure 1). There were 181 male (29%) and 454 female (71%) respondents; 167 freshmen (26%), 114 sophomores (18%), 120 juniors (19%), 143 seniors (22%), 16 fifth-year students (3%), and 75 postgraduates (12%). 309 (49%) students specializing in clinical medicine, 238 (37%) from the nursing specialty, and 88 (14%) from other medical specialties. 97 (15%) had no siblings and 537 (85%) had siblings. 144 (23%) had experience of being a class leader or were a class leader, and 492 (77%) did not or were not. 151 (24%) participated in prevention and control of the epidemic, and 484 (76%) did not. 113 (18%) were from cities, 135 (21%) were from towns and 387 (61%) were from rural areas.

#### Method

With reference to the medical students’ professional identity questionnaire prepared by Fujian Medical University, the questionnaire was increased and decreased based on the research problem. Except for the professional behavior dimension, the professional environment, the professional value, professional cognition, professional emotion, and professional expectations dimensions were selected and scored using a 5-point system. The higher the average score, the higher a person’s sense of professional identity. Self-designed questionnaire concluding the questions: Do you think choosing a medical specialty is right (1 yes, 2 no)? Is your decision to be a physician in the future firm (1 yes, 2 no)? Do you think that medical science is a sacred and worthy career (1 yes, 2 no)? which assessing their professional attitude after COVID-19. These were scored using a 2-point scoring system, the lower the average score, the higher a person’s professional attitude.

### Statistical Method

SPSS25.0 software package was used for the data analysis.

## Results

### General Status of Medical Students’ Professional Identity

**FIGURE 2:**
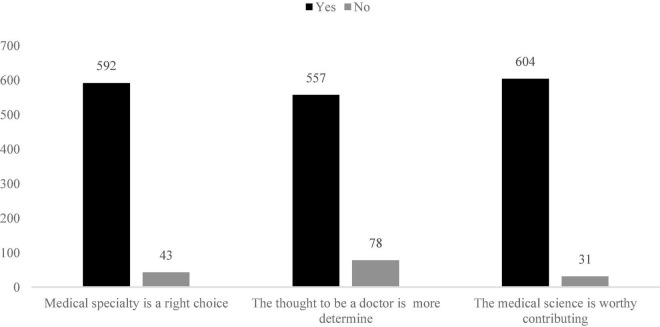
Medical students’ professional attitude after COVID-19.

According to Table 1, the total average score of medical students’ professional identity is 3.39, which is above average. The average score of each dimension is between 3.02-3.72, which is the upper middle level. Medical student has the highest score in terms of professional expectation identity and the lowest in professional environment identity. According to the standard deviation (*SD*) in the results, the biggest difference is with professional expectation which is 0.67, this indicates that medical students make extreme choices in terms of their career expectations. Most students think that choosing a medical specialty is right (93%), the determination of doctors is more determined (88%), and that medical science is worthy contributing to (95%) since the outbreak of COVID-19. These are shown in Figure 2.

**TABLE 1:** Analysis of 635 medical students’ professional identity.

	P-En	P-Va	P-Co	P-Em	P-Ex	Total
M	3.02	3.35	3.12	3.67	3.72	3.39
SD	0.50	0.58	0.43	0.64	0.67	0.39

*P-En = Professional environment, P-Va = professional value, P-Co = Professional cognition, P-Em = professional emotion, P-Ex = Professional expectation.*

### Single Factor Analysis of Medical Students’ Professional Identity

#### the Gender Differences in Medical Students’ Professional Identity

An independent sample *t*-test was used to explore the impact of gender on medical students’ professional identity. The results (Table 2) show that boys scored higher than girls in the dimensions of professional value, professional emotion, and professional expectation, but only the dimension of professional value had significant differences. The girl’s scores were significantly higher than boys in the professional environment and professional cognition dimensions. There were no significant differences in professional emotion, expectation, and total average scores.

**TABLE 2:** Analysis of gender differences in medical students’ professional identity.

	M (*n* = 181)	F (*n* = 454)	t	*p*
P-En	2.90 ± 0.49	3.07 ± 0.49	−3.918	0.000***
P-Va	3.42 ± 0.58	3.32 ± 0.59	2.057	0.040*
P-Co	3.02 ± 0.46	3.16 ± 0.41	−3.855	0.000***
P-Em	3.74 ± 0.67	3.65 ± 0.63	1.658	0.098
P-Ex	3.80 ± 0.67	3.68 ± 0.66	1.861	0.063
Total	3.40 ± 0.42	3.40 ± 0.37	0.198	0.843

*P-En = Professional environment, P-Va = professional value, P-Co = Professional cognition, P-Em = professional emotion, P-Ex = Professional expectation.*

* = p < 0.05, *** = p < 0.001.

#### The Impact of One-Child Status on Medical Students’ Professional Identity

The results (Table 3) show that the scores for people without siblings (only children) were higher than those for children with siblings, except for the professional environment dimension. The differences in professional emotion and expectation dimension were significant. The average score for professional identity among only children was significantly higher than among children with siblings.

**TABLE 3:** Influence of only children or not status on medical students’ professional identity.

	Only (*n* = 97)	Non-only (*n* = 538)	t	*p*
P-En	3.02 ± 0.54	3.02 ± 0.49	0.055	0.957
P-Va	3.40 ± 0.67	3.34 ± 0.57	0.805	0.422
P-Co	3.18 ± 0.40	3.11 ± 0.43	1.403	0.161
P-Em	3.80 ± 0.69	3.65 ± 0.63	2.150	0.032*
P-Ex	3.86 ± 0.68	3.69 ± 0.66	2.349	0.019*
Total	3.47 ± 0.40	3.38 ± 0.38	2.264	0.024*

*P-En = Professional environment, P-Va = professional value, P-Co = Professional cognition, P-Em = professional emotion, P-Ex = Professional expectation.*

* = p < 0.05.

#### The Impact of Participated in Prevention and Control of the Epidemic on Medical Students’ Professional Identity

The results (Table 4) show that the scores of medical students who participated in prevention and control of the epidemic were higher than those who did not, this was true for the professional value, cognition, emotion and expectation dimensions, the differences were significant except for the professional value. Medical students who did not participated scored higher in the professional environment, but the difference was not significant. Overall, the professional identity scores of participated medical students were significantly higher.

**TABLE 4:** Analysis of the differences between participated or not.

	Participate (*n* = 151)	No participate (*n* = 484)	t	*p*
P-En	2.96 ± 0.52	3.04 ± 0.49	−1.675	0.094
P-Va	3.41 ± 0.64	3.33 ± 0.57	1.455	0.146
P-Co	3.18 ± 0.48	3.10 ± 0.41	2.076	0.038*
P-Em	3.81 ± 0.70	3.63 ± 0.62	3.107	0.004**
P-Ex	3.81 ± 0.66	3.69 ± 0.67	1.932	0.054*
Total	3.46 ± 0.44	3.37 ± 0.36	2.267	0.009**

*P-En = Professional environment, P-Va = professional value, P-Co = Professional cognition, P-Em = professional emotion, P-Ex = Professional expectation.*

* = p < 0.05, ** = p < 0.01.

#### The Grade Differences of Medical Students’ Professional Identity

The results of one-way ANOVA show that medical students’ professional identity gradually declined from freshman year to fifth grade before increasing at the graduate stage. The differences between the grades were significant [*F*_(5,635)_ = 10.302, *p* < 0.001]. *Post hoc* multiple comparative analysis show that the significant difference came from freshman, sophomore, junior, senior and graduate grades. See Tables 5, 6.

**TABLE 5:** Medical students’ professional identity scores in different grades.

	N	Total average scores
Freshman	167	3.56 ± 0.32
Sophomore	114	3.37 ± 0.35
Junior	120	3.35 ± 0.42
Senior	143	3.29 ± 0.40
fifth year of college	16	3.26 ± 0.26
Graduate	75	3.33 ± 0.40

*F = 10.302 p < 0.001.*

**TABLE 6:** Multiple comparative analysis of medical students’ professional identity in different grades.

	1	2	3	4	5
1. Freshman					
2. Sophomore	0.003**				
3. Junior	0.001***	1.000			
4. Senior	0.000***	0.681	0.828		
5. Fifth year of college	0.094	0.946	0.971	1.000	
6. Graduate	0.001***	0.990	0.999	0.988	0.995

** = p < 0.01, *** = p < 0.001.

#### The Professional Differences of Medical Students’ Professional Identity

The results of one-way ANOVA show that the clinical scores were highest and the scores of others were lowest. There were significant differences among majors [*F*_(2,635)_ = 5.718, *p* < 0.01]. The results of *post hoc* multiple comparative analysis showed that there were significant differences between clinical and nursing specialties and others, but there was no significant difference between nursing and others (see Tables 7, 8).

**TABLE 7:** Comparison of medical students’ professional identity in different specialties.

	N	Total average scores
Clinical	309	3.44 ± 0.37
Nursing	238	3.35 ± 0.38
Others	88	3.33 ± 0.44

*F = 5.718, p < 0.01.*

**TABLE 8:** Multiple comparative analysis of different medical specialty.

	1	2
1. Clinical		
2. Nursing	0.016*	
3. Others	0.038*	0.890

* = p < 0.05.

### Multi Factor Analysis of Medical Students’ Professional Identity

Taking students’ total average scores as the dependent variable, the 10 factors that may affect professional identity: gender, grade, specialty, registered residence, being an only child or not, being a class leader or not, being participated in prevention and control of the epidemic or not, occupational attitudes after COVID-19, college choosing, and family member’s influence as the independent variables. As shown in Table 9, family member’s influence, professional attitudes after COVID-19, grades, specialty, registered residence, and college choosing were the main factors that influenced medical students’ professional identity. Gender, siblings, class leadership experience, and participation had little effect. Students who had a positive attitude toward the medical career after COVID-19 had higher level of professional identity. Medical students with lower grades had a greater sense of professional identity than students with higher grades. Professional identity was higher among students from cities than students from rural areas, and higher among first choose than non-first choose. See Table 10.

**TABLE 9:** Assignment of variables affected medical student’s professional identity.

Variables	Assignment
Total scores	Number
Professional attitude	Number
Gender	1 M;2 F
Only Child	1 Yes;2 No
Grade	1 Freshman;2 Sophomore;3 Junior;4 Senior;5 fifth year of college;6 Graduate
Specialty	1 Clinical;2 Nursing;3 Others
Registered residence	1 City;2 Town; 3 rural areas
Class leader	1 Yes;2 No
Participated in	1 Yes;2 No
College choosing	1 First;2 Second;3 Third and later;4 Adjustment
Family member’s influence	1 Great;2 Little;3 No

**TABLE 10:** Multi influence factor analysis of medical students’ professional identity after COVID-19.

Predictive variable	B	SD	Beta (β)	t
Constant	4.568	0.122	−	37.386***
Gender	0.043	0.031	0.050	1.388
Only Child	–0.023	0.042	–0.021	–1.241
Participate in or not	–0.050	0.034	–0.056	–1.473
Class leader or not	–0.056	0.029	–0.072	–1.925
Family’s influence	–0.039	0.019	–0.076	−2.066***
Professional attitude after COVID-19	–0.544	0.066	–0.308	−8.291***
Grade	–0.041	0.009	–0.173	−4.633***
Specialty	–0.049	0.020	–0.091	−2.473*
Registered residence	–0.046	0.020	–0.093	−2.354*
College choosing	–0.027	0.013	–0.077	−2.106*

R = 0.465 R^2^ = 0.216 After adjustment R^2^ = 0.204 F = 17.230***. * = p < 0.05, *** = p < 0.001.

## Concluding Remarks

Medical education is an important cornerstone of a career in health care. Many high-quality medical talents are educated based on a vigorously developed medical education system. Medical students’ professional identity is related to their career development and the realization of the Healthy China Strategy. It is of great educational and social significance to strengthen their thoughts about their profession and improve their professional identity.

The survey found that medical students’ professional identity is higher since the outbreak of COVID-19. It is above average level, which shows that more people now understand the importance of medicine. The professional spirit of being unafraid of difficulties and dangers, saving the dying, healing the wounded, and being benevolent during the prevention and control of the epidemic have all affected medical students’ understanding of their professional identity. There are some differences between specialty, grades, and location, all of which chime with previous studies. The reasons for this may be related to the unequal resources present in cities and rural areas or due to the economic or educational situation of families, students’ different understandings of specialties, their employment prospects, and the environment. For example, most people may first respond to clinical specialties when they mention the word “doctors,” but students in many other medical specialties can become doctors in the future, including pediatrics, stomatology, anesthesia, and psychology students (Zheng et al., 2021). Professional identity gradually decreases with grades, the possible reasons for this may be that the medical learning cycle is long, the academic demands are significant, and there is a large amount of pressure (Zhang and Ma, 2016). Also, social media and public opinion present exaggerated negative reports about the doctor-patient relationship and the medical environment (Du, 2014). Therefore, medical students’ professional environment and expectation decrease as they gain a deeper understanding of the professional value and social status of doctors, but their professional identity also increases in the graduate stage. There were many student volunteers who worked during COVID-19. Participating in the prevention and control of the epidemic provided students with personal experience of working as a professional health care provided (Park et al., 2020). The study found that medical students who participated in prevention and control of the epidemic had significantly higher levels of professional identity. However, this was not the main factor that affected their professional identity. People now have a deeper understanding of medical science after COVID-19, and medical students have a positive attitude toward studying medicine and becoming a doctor, these are the main factors affecting professional identity. The influence of family and college choosing are others main factors.

### Suggestions

1. Strengthen the focus on professionalism among medical students. Use freshmen entrance educations, secondary classroom activities, and lectures by famous doctors or teachers to integrate thinking about the profession into the curriculum. This will help to deepen medical students’ understanding of their specialty and improve their professional identity.
2. Increase education about career planning for medical students. Career planning should run from the admissions stage to the internship stage and the training stage. This should help students to understand the training objectives and professional ethics of the medical profession. This should stimulate student’s internal drive to study actively, enhancing their ideals and beliefs, and strengthen their determination to become a doctor in the future.
3. Carry out more teaching for clinical skill training and social practice. Clinical skill training is important for forging students’ and workers’ professional identity. More attention had paid to the cultivation of professional ability and the assessment of it during clinical skill training. This will ignore ideological education (Bridges et al., 2011). Therefore, school teaching should involve social practice education that combines theory with practice to improve students’ professional identity. Students’ performance should be strictly assessed during the clinical skill training stage. This should involve testing not only skills but also students’ professional ethics and the ideological aspects of clinical skill. Close attention should be paid to this important period and its influence on medical students’ professional identity (Cruess et al., 2015).

## Data Availability Statement

The raw data supporting the conclusions of this article will be made available by the authors, without undue reservation.

## Ethics Statement

This study was reviewed and approved by the Academic Ethics Committee of Gannan Medical University. Written informed consent from the patients/participants or patients/participants legal guardian/next of kin was not required to participate in this study in accordance with the national legislation and the institutional requirements.

## Author Contributions

TL reviewed the literature and wrote the manuscript. WL, MZ, and PZ contributed to data collection. BL edited the manuscript. All authors contributed to the article and approved the submitted version.

## Conflict of Interest

The authors declare that the research was conducted in the absence of any commercial or financial relationships that could be construed as a potential conflict of interest.

## Publisher’s Note

All claims expressed in this article are solely those of the authors and do not necessarily represent those of their affiliated organizations, or those of the publisher, the editors and the reviewers. Any product that may be evaluated in this article, or claim that may be made by its manufacturer, is not guaranteed or endorsed by the publisher.
